# A Dataset for Victim Detection in Search-and-Rescue Operations Using Robot-Mounted UWB-Radar Sensors

**DOI:** 10.1038/s41597-026-07314-z

**Published:** 2026-05-12

**Authors:** Antonios-Periklis Michalopoulos, Efstratios N. Paliodimos, Fotios Papadopoulos, Grigoris Nikolaou, Charalampos Z. Patrikakis, Stylianos A. Mytilinaios

**Affiliations:** 1 Department of Electrical and Electronics Engineering, University of West Attica, Athens, Greece; 2 Department of Industrial Design and Production, University of West Attica, Athens, Greece

**Keywords:** Electrical and electronic engineering, Scientific data

## Abstract

Search-and-Rescue (SAR) operations rely on sensor platforms to detect victims behind walls, obstacles etc. Conventional sensors (thermal, optical etc.) face limitations in occluded environments, whereas Ultra-Wideband (UWB) radar can penetrate obstacles, offering a promising solution for victim detection. Meanwhile, robotic platforms can significantly enhance disaster response operations, especially in hazardous environments. This paper introduces a comprehensive dataset of victim measurements from a robot-mounted radar sensor. The dataset design closely emulates SAR scenarios inside buildings for victim detection and localization. To the best of our knowledge, this is the first dataset capturing robot movement during SAR operations and focusing on UWB radar sensing from multiple orientations. To demonstrate the technical validity of the proposed dataset alongside its applicability in supporting further development of Artificial Intelligence (AI) and Machine Learning (ML) models, we also present a CNN model and an XGBoost model for victim detection, achieving F1-scores of 78% and 83% respectively. Moreover, a rule-based position estimation method achieved a Mean Absolute Error (MAE) of 0.49 m, further highlighting the dataset’s value.

## Background & Summary

Rapid response is critical in Search-and-Rescue (SAR) missions, as it directly affects a victim’s chances of survival^1^. First responders (FRs) frequently encounter challenges in locating individuals who are trapped behind walls, rubble or other obstacles, where conventional sensing modalities like thermal or acoustic sensors often prove ineffective. Radar technology has emerges as a promising alternative due to its ability to penetrate various materials and detect vital physiological signals. Notably, the periodic motion of the torso due to breathing can be captured by radar systems since it is related to a similarly periodic change of the body’s average dielectric constant. At the same time, robotic platforms can significantly support FRs by facilitating remote sensing and action, thus reducing the risk of human exposure in hazardous environments^2^. Integrating radar systems with robotic platforms further enhances SAR operations by enabling autonomous victim detection and localization, and improving SAR efficiency.

Currently, there are limited examples of robotic systems equipped with radar-based sensors, as well as relevant datasets, and even fewer instances of radar data from robotic platforms capable of detecting and locating victims behind walls. Some studies focus on radar-based robotic systems^3^ but these approaches often require an unobstructed line of sight to the individual for effective radar operation^4–6^. Rohman *et al*.^7^ improved upon their previous work^5^ by exploring the use of UWB radar mounted on Unmanned Aerial Vehicles (UAVs), to locate individuals in more complex scenarios through extensive signal processing. Schroth *et al*.^8^ presented a similar approach using an autonomous robotic system and advanced signal processing to estimate a person’s distance behind obstacles. In addition, certain studies have utilized radar sensors for detecting breathing patterns behind obstacles^9^ and foliage^10^, but there is an apparent lack of publicly available datasets that integrate such measurements in dynamic, robot-assisted scenarios. Study^9^ employed the X4M200 ultra-wideband radar sensor (Novelda) in a static environment, with the radar positioned parallel to the subject. In contrast, study^10^ adopted an identical radar sensor configuration to that used in the present work, while incorporating dynamic obstructions, specifically foliage. However, the radar system itself remained static and was oriented parallel to standing subjects. Our proposed dataset addresses this gap and offers a flexible benchmark for developing and evaluating robust radar perception algorithms.

Indeed, our key difference with respect to^8–10^, or other literature, is that we propose a comprehensive dataset that is designed to support and enhance the development of automated robotic systems equipped with radar sensors for human detection in SAR operations. We build upon our previous work^11^ to craft an extended, comprehensive dataset. Unlike previous works that focus on static setups and limited scenarios, this one captures radar data from multiple positions and angles, thus providing rich spatial diversity. Indeed, our core scenario is that of a robot that systematically scans an indoor environment and searches for victims hidden behind walls or obstacles. This data variability enables researchers to explore distance estimation, movement detection, and localization tasks under realistic conditions. This, together with the proposed dataset’s technical validity, showcased by a toolset of techniques for victim detection and localization (including machine and deep learning models), lays a strong foundation for facilitating further research in the field of automated, robot-enhanced SAR operations.

In the following, Section “Methods” introduces the proposed robot-mounted radar platform with the sensors and the methodology used to collect the data, Section “Data Records” presents the developed dataset and provides a data overview, Section “Technical Validation” showcases the technical validity of the proposed dataset in terms of its applicability for the development of machine and deep learning models and, finally, Section “Usage Notes” offers suggestions for further utilization of the data, including potential extensions with non–machine learning approaches.

## Methods

### Participants and ethical requirements

The identities of the human subjects (victim actors) that participated in the data collection process were pseudo-anonymized using number IDs instead of names or emails. Demographic data of participants are discussed in Dataset development methodology below. The study was approved by the Research Ethics Committee of the University of the West Attica (Approval No. 50150, 28/06/2021). Additionally, it should be noted that all participants signed a written informed consent for the collected data to be published.

### Experimental setup and robotic sensor platform

**Fig. 1:**
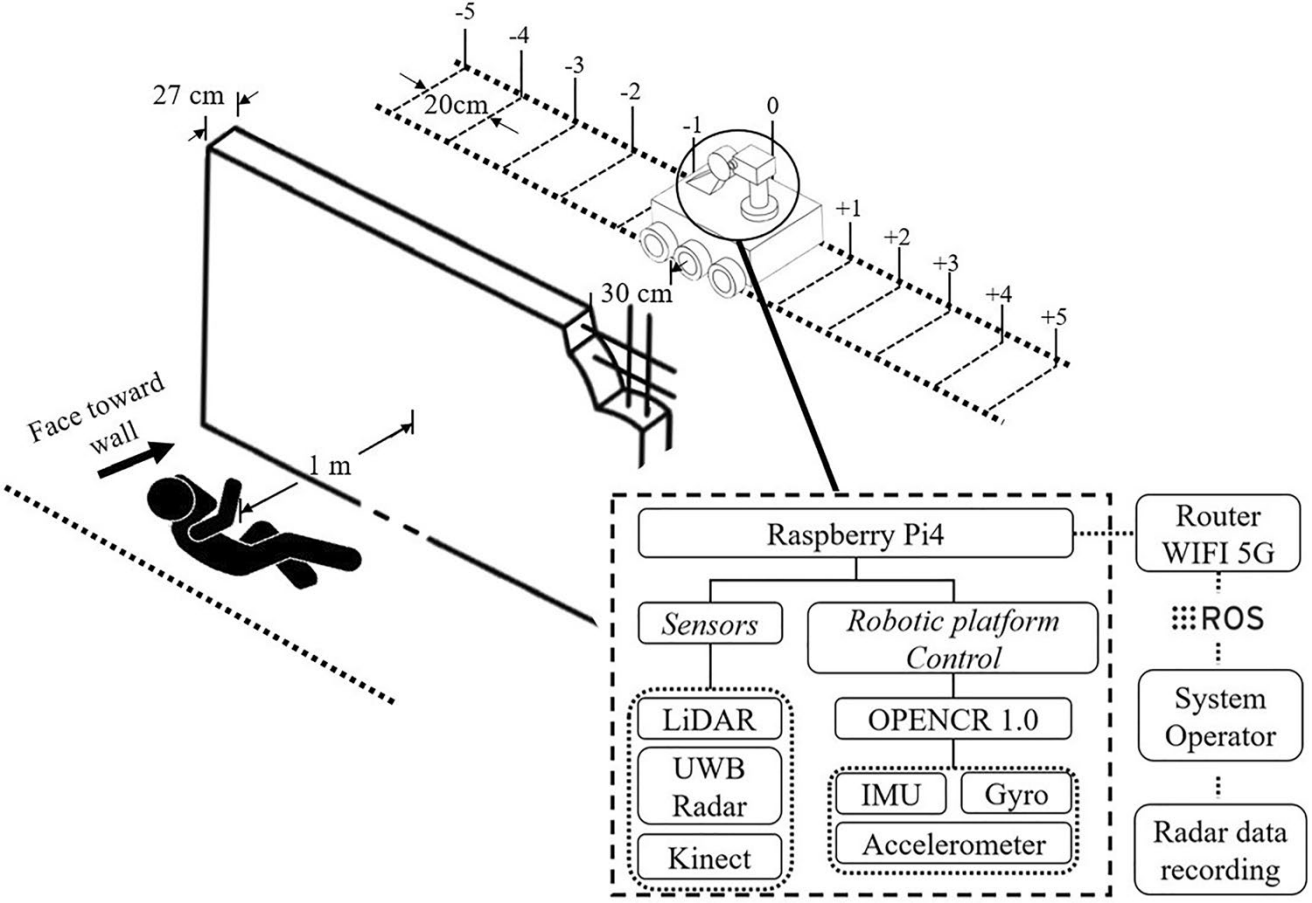
Dataset collection setup and robotic platform components.

The proposed experimental setup is illustrated in Fig. 1. The scenario that we emulate with our experimental setup is that of a robot that is autonomously scanning an indoor area during the aftermath of a crisis and is searching for victims that are hidden by walls, trapped behind obstacles, etc. As implied by Fig. 1, and according to the emulated scenario and use case, the robot autonomously navigates along the perimeter of indoor walls while scanning for victims trapped behind these walls. Furthermore, it does so while keeping a constant distance from the wall(s) and performing turns and maneuvers either autonomously or remotely controlled.

In order to perform victim scans and navigate around the area, the robotic platform is equipped with an Ultra-Wideband (UWB) radar sensor, model SLmX4 by SensorLogic^12^, an odometer sensor, a LiDAR (Light Detection and Ranging) sensor, type LDS-01^13^ and a legacy Kinect V1 dual-camera sensor by Microsoft. In reverse order, the Kinect V1 camera is used for remote scene surveillance and stores images and video at 30 frames per second (fps) with a resolution of 640 × 480 pixels while performing depth estimation up to 5 meters. The LiDAR sensor is used by the robot for autonomously controlling its route by keeping a constant distance of 30 cm to the wall (see Fig. 1), while the odometer sensor allows for constant pace displacements of the robot (in steps of 20 cm according to Fig. 1), as it scans for victims. The robotic platform is remotely controlled via WiFi by a PC running the Robot Operating System (ROS), while an onboard edge computing unit (Raspberry Pi 4) controls the robot movement, the sensors and the data acquisition procedure, and the wireless connectivity in real-time with the remote PC.

As long as the radar sensor is concerned, it is built around Novelda’s X4 chip^14^ and operates at either the 7.29 or 8.74 GHz center frequency with a bandwidth of 1.4 GHz. Its antenna configuration enables wide-angle coverage with 3 dB beamwidths of 87.5°(elevation) and 114.3° (azimuth) while its operational parameters are listed in Table 1. The radar sensor is installed on the side of the robot (see Fig. 1), with a 40 × 25 cm rectangular metal reflector placed 8.4 cm behind the radar antenna and between the sensor and the rest of the robotic platform; the reflector shape, dimensions and position were selected for optimal rejection of radar clutter introduced by the robotic platform to the sensor.

**Table 1:** Radar configuration.

Parameter	Value	Parameter	Value
Pulses per second (PPS)	1200	Frames per second (FPS)	19
Center Frequency (GHz)	7.29	Iterations	4
Transmit Power (dBm)	6.3	Number of samples (range bins)	180
Resolution (m)	0.0512	DAC range (a.u.)	949–1100
Pulse repetition frequency	16	Reflector position	2λ (8.4 cm)
(MHz)			

To enable the integration of the SLMX-4 radar module within our robotic platform, we developed a dedicated Python library^15^ derived from the original MATLAB codebase provided by Sensor Logic^16^. This transition to Python was essential for ensuring compatibility with embedded systems, such as the Raspberry Pi 4 (2GB model) used in our robot. Additionally, this library serves as the backbone of a custom ROS (Robot Operating System) node, acting as an interface between the radar-equipped robotic system (acting as the slave) and an external laptop (acting as the master). This architecture supports continuous data acquisition and immediate interpretation, enabling real-time feedback and responsive decision-making during robotic operation.

### Data acquisition and data format

**Fig. 2:**
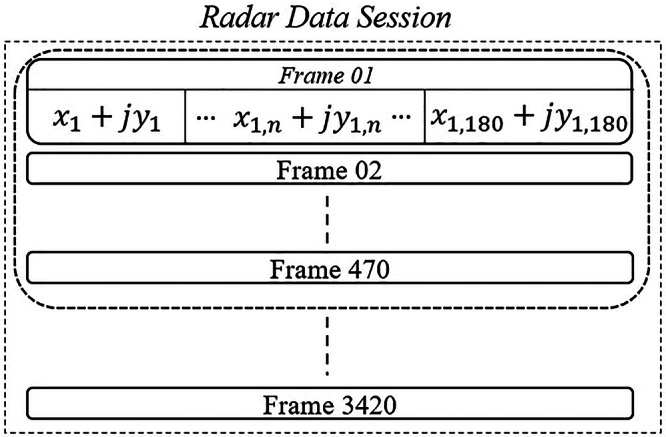
Radar data session structure.

The radar measurements are collected during controlled robotic experiments, as depicted in Fig. 1. The robot is programmed to follow a linear trajectory in a laboratory environment, moving along a pre-defined straight line in discrete steps of 20 cm, with a reference origin marked as position 0. The robot traverses a total of 11 positions (from −5 to +5), spanning a total length of 2 meters. At each position, the robot remains stationary and records a sequence of ~3420 radar *frames* (180 s duration), forming a so-called radar *session* (see Fig. 2 below). Sessions from different robot positions are collected, for different human subjects lying down at different distances from the wall (1 m in Fig. 1) and various torso orientations (body facing the wall in Fig. 1), resulting in a high-dimensional spatiotemporal dataset.

By reference to Fig. 2, a radar data session is a sequence of 3420 radar frames with *i* being the row index. Each frame corresponds to a single radar measurement that returns a row vector of 180 cells, which corresponds to the complex envelope of the radar backscattered signal. More specifically, within each frame the radar data are represented as a one-dimensional array of complex-valued samples where n = 1…180 denotes the range bin index that maps propagation delay to physical range. These complex values represent the baseband complex envelope of the radar’s received signal, encoding both amplitude and phase information essential for coherent radar processing. Also, with a range resolution of 0.0512 m (see Table 1), the range of each radar measurements is calculated to be equal to about 9.2 m.

With a rate of around 19 fps (see Table 1), it follows that each radar session corresponds to about 180 seconds of measurement time. Moreover, consecutive rows within each session are combined to form data *samples*. As detailed in Section 4 (Technical Validation) of this paper, a sample size of 470 frames is selected; this is illustrated for the first sample of a session in Fig. 2. The rationale for the number 470 is that, with an average human breathing cycle duration of 4–6 s, each sample is expected to include a total of 4–6 breathing cycles.

With respect to dataset development, we collected a large number of radar sessions for each robot position, human subject, human-robot distance and human orientation to the wall. The specifics of the dataset development methodology are discussed in the following Section.

## Data Record

The dataset is available online at Zenodo, under the name “A Comprehensive Dataset for Victim Detection in SAR Operations Using Robot-Mounted UWB-Radar Sensors”^17^, with this section being the primary source of information on the availability and content of the data being described.

### Dataset development methodology

Six human subjects, acting as “victim” actors, participated in the dataset development representing a diverse demographic profile in terms of age, sex, height, weight, and other physical characteristics (see Table 2). The data acquisition was conducted at the Antenna Laboratory of the Department of Electrical and Electronic Engineering, University of West Attica (UNIWA). By reference to Fig. 1, each subject was positioned behind a 27 cm-thick YTONG wall, at two distinct distances from the wall—1 meter and 2 meters—and in four different body orientations: supine (face up), prone (face down), facing toward the wall, and facing away from the wall. For each configuration, radar measurements were collected from 11 discrete robot positions, uniformly spaced at 20 cm intervals, providing systematic spatial coverage over a total span of 2 meters. The robot platform is initially positioned at a distance of 30 cm from the wall and is automatically moving to the next position as soon as a radar session measurement is completed. To ensure precise displacement and repeatability across measurement positions, the robot’s onboard odometer and LiDAR sensors were employed for localization and motion tracking: the odometer sensor ensured repeatability of position-to-position displacement while the LiDAR sensor ensured a constant distance between the robot and the wall. The complete dataset generated through this procedure is made publicly available in^17^.

**Table 2:** Participants’ data.

ID	Gender	Height (cm)	Age	Weight (Kg)	Activity
1	Male	172	25	75	Medium
2	Male	195	24	114	Low
3	Male	178	24	101	Low
4	Female	150	23	54	High
5	Female	167	22	58	Medium
6	Male	172	26	78	High

The dataset may be considered a collection of human breathing pattern measurements, but its actual objective is broader. Thanks to the mobility and precision of the robotic system, we were able to collect radar measurements from multiple angles and positions relative to the same subject in a fast and repeatable manner. As a result, the dataset is best described as a multi-angle human detection dataset, offering a structured and diverse set of observations that reflect varying orientations, distances, and sensor viewpoints. This capability is particularly valuable for training and evaluating detection models that need to generalize across different spatial configurations, as encountered in real-world robotic and search-and-rescue scenarios.

This multi-angular nature of the dataset introduces a notable side effect: changes in the effective radar-to-target distance caused by angular displacement. As the robot moves along the wall, the radar signal encounters the subject at progressively steeper angles, altering the signal’s travel path and its corresponding effective propagation velocity and subsequent range estimation. To better understand these effects, we present the results of a side experiment to demonstrate how the radar-to-target measured distance changes at each robot position. Following the setup of Fig. 1, the experiment was conducted under the same conditions as the main dataset, using the same YTONG wall and setup geometry. The “target” was placed 1 meter behind the wall, while the robot maintained a fixed distance of 30 cm from the wall. Given the wall thickness of 27 cm, the total distance between the radar and the subject when the robot was in the central position (P₀) was 1.57 meters. Then, we compare the distances reported by the radar at different positions (from −5 to +5, see Fig. 1) with the actual Euclidean distances between the radar and the subject calculated using the Pythagorean theorem. These results are tabulated in Table 3, where P_i_ is the horizontal displacement of the robot relative to the center point 0 (in centimeters), *d*_*i*_ is the actual Euclidean distance between the radar and the subject and ${\hat{d}}_{i}$ is the distance estimate returned by the radar. The differences between d_i_ and ${\hat{d}}_{i}$ reveal the distortion introduced by the change of the radar-measured delay of the backscattered signal. For oblique angles, the radar signal travels for a longer time inside the wall, resulting in both a weaker signal and a distorted delay introduced by the material’s dielectric constant compared to the dielectric constant of the air.

**Table 3:** Effect of signal travel path to radar-estimated target distance due to changes in the signal travel path through the wall (*P*_i_: horizontal displacement of the robot relative to the center point 0 in centimeters; *d*_*i*_: the actual Euclidean distance between the radar and the subject; ${\hat{d}}_{i}$ the distance estimate returned by the radar).

Radar position	*P*_*i*_ (cm)	*d*_*i*_ (cm)	${\hat{{\boldsymbol{d}}}}_{{\boldsymbol{i}}}$(cm)
0	0	157	157
±1	20	158	162
±2	40	162	170
±3	60	168	185
±4	80	176	200
±5	100	186	210

### Dataset folder structure

**Fig. 3:**
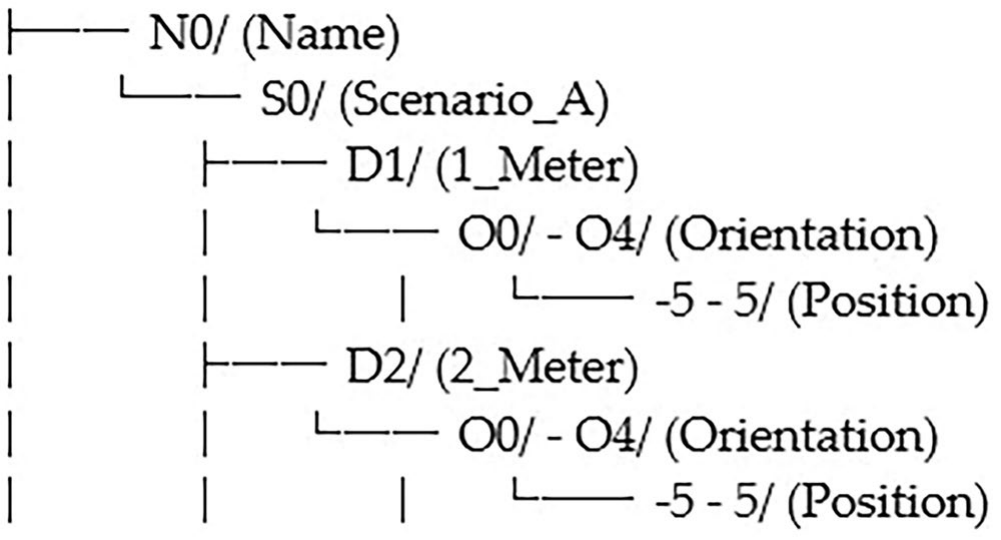
Dataset folder structure.

The dataset includes two main data classes: Presence and Absence of human subjects within the radar’s field of view. The Absence class includes data recorded when no subject was present and is represented by entries where the subject ID, denoted by “N”, is set to N0. These recordings vary only by the robot’s relative position, denoted by “X”, ranging from −5 to 5 (as in Fig. 1), without additional distinctions e.g. in terms of distance or orientation. In contrast, the Presence category is fully defined by a structured code N_S_D_O_X, which captures all relevant experimental parameters. Specifically, the codename N_S_D_O_X fully characterizes the data included in the first column on each available CSV with the name of “N”. More specifically, each human subject is labeled with a unique identifier Ni with i ranging from 1 to 6. The session number S is fixed at 1 for all recordings. The distance from the wall (D) can be either 1 or 2 meters. The subject’s orientation is encoded in O using the following mapping: face toward the wall as O1, face away as O2, face up as O3, and face down as O4. Finally, the robot’s position relative to the subject is denoted by X, with values ranging from −5 to 5. A visual representation of the dataset folder structure is illustrated in Fig. 3.

All available data from each subject was consolidated into a corresponding CSV file named data_Ni.csv, where i denotes the subject ID, ranging from 0 (for Absence) to 6. For better usability, the dataset is provided in two formats. The first is a single, unified CSV file containing all available data across subjects and conditions, and due to the substantial size of this consolidated file, it is made available upon request to the authors. The second format includes individual CSV files (N1.csv to N6.csv for Presence, and N0.csv for Absence), allowing more targeted access to specific sessions or subject configurations as the single CSV size is about 18 GB.

The structure within each CSV file consists of five columns containing numerical data. The first column, ID_code, uniquely identifies each sample using the format N_S_D_O_X, encapsulating the subject ID, session, distance, orientation, and robot position. The second column records the Elapsed_time of each measurement in seconds since the beginning of the session. The third column, Complex_data, contains an array of 180 complex numbers, representing the radar signal for the current frame. The final two columns include annotations: Presence, indicating whether a human subject was present (1) or absent (0), and Distance, specifying the distance between the robotic system and the subject during that session.

### Data overview

In total, each subject contributed 264 minutes of recordings, which equals approximately 4.4 hours of data or 88 sessions of 3 minutes. With a frame rate of 19 frames per second, this results in 300,960 rows per person. Looking at it from the perspective of each unique robot position, the dataset includes around 2.4 hours of data per position, equivalent to 164,160 rows, captured at the same frame rate. This provides a substantial amount of data for each configuration, supporting thorough and meaningful analysis. Minor variations of approximately ±0.2 FPS were observed across sessions resulting in slightly different numbers of rows and seconds, but all staying around the corresponding position of 19 FPS for 180 seconds. At the same time, the absence data was collected following the same setup and principles. However, since no “victim” was present, each position was recorded twice to ensure consistency. Each absence session lasted 30 minutes, resulting in approximately 34,200 rows of data per position or 5.5 hours. As a result, the data that has been collected corresponds to 2,179,281 rows of absence and presence, with presence having 1,806,240 and absence 373,041 making the dataset in total approximately 17 GB and almost 32 hours.

**Fig. 4:**
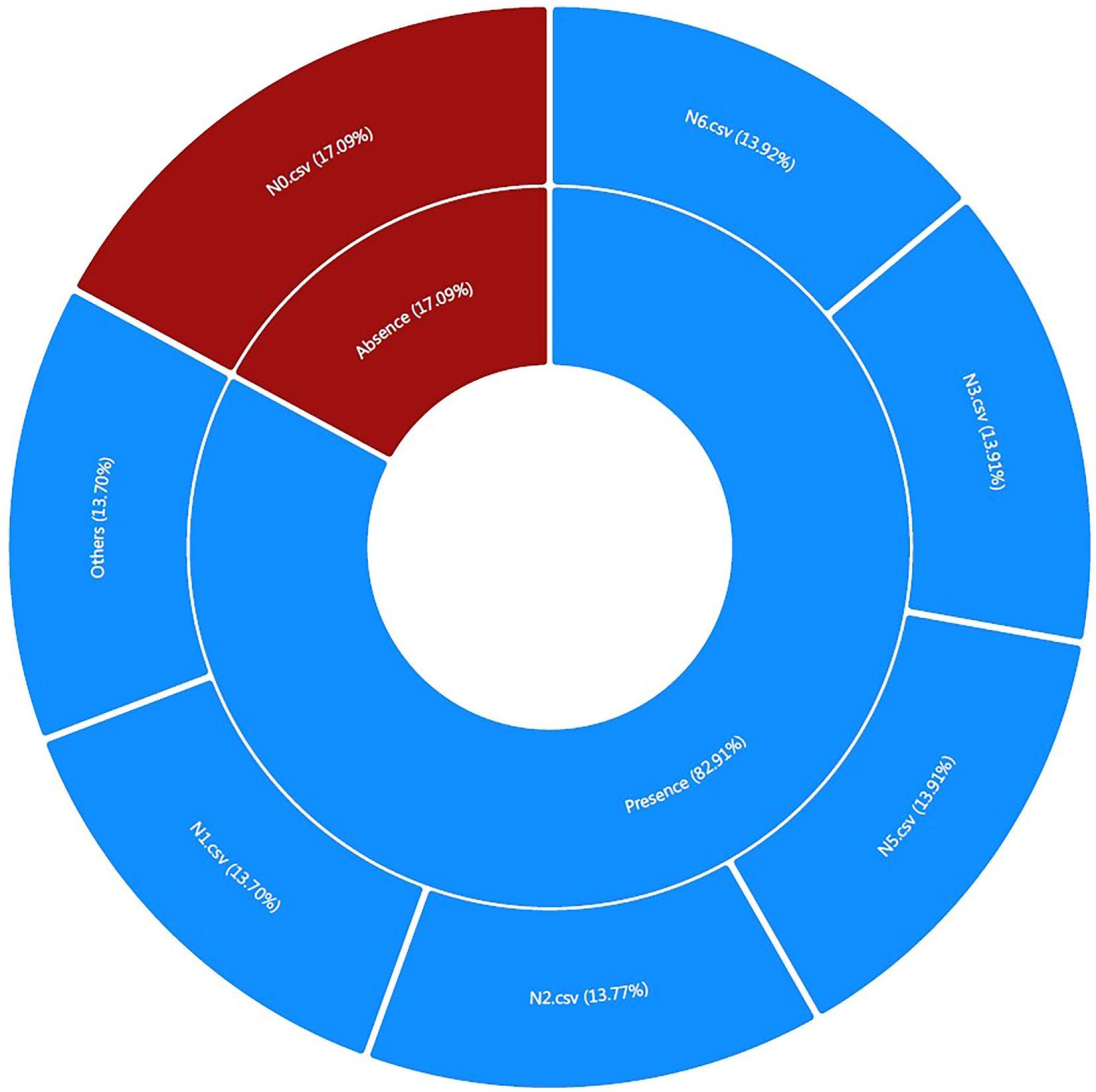
Visual representation of recorded sessions percentages for Presence and Absence.

Figure 4 illustrates the share of each subject, as well as the absence data, in terms of total recorded duration, number of CSV lines, and file size in GB. As clearly shown in Fig. 4, the dataset is imbalanced towards the presence class, with approximately 83% of the data labeled as presence. This is expected, since the primary focus of the dataset is human detection. The remaining 17% corresponds to the absence class, far from perfect ratio but still within an acceptable range. That said, there are several ways the absence class can be augmented without the need to record new sessions. Some examples of potential augmentation techniques are discussed in the Usage Notes section of this paper.

**Fig. 5:**
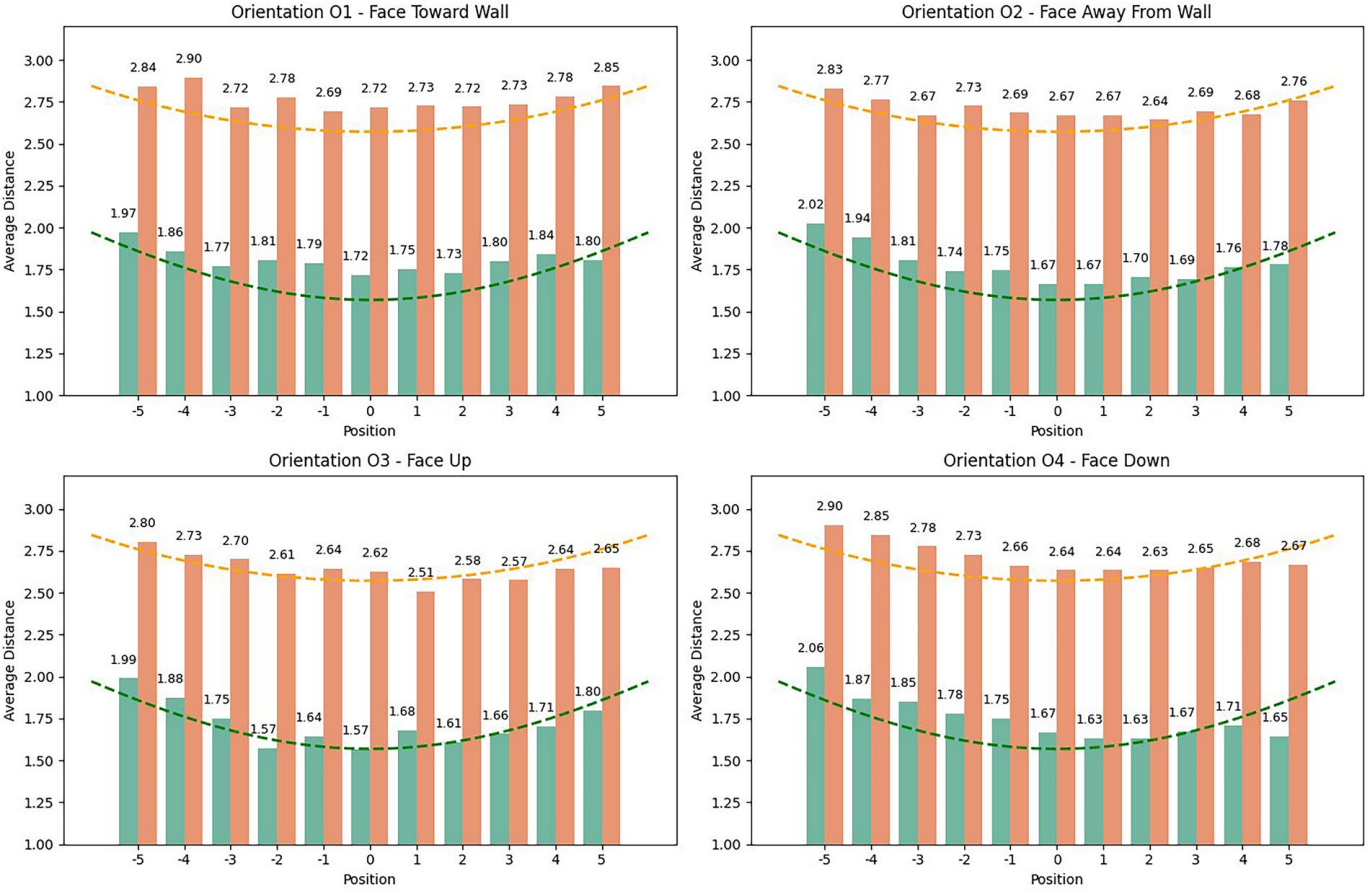
Annotation distance vs. Euclidean distance deviation.

To further explore the dataset, we analyzed the deviation between the estimated radar-to-target distance after each human annotation in a session and the actual Euclidean target distance. The deviations observed, especially those illustrated in the boxplots of Fig. 5 can be attributed to distortion caused by the altered signal path, as discussed in Table 3 and detailed in Subsection 3.1. This effect is particularly noticeable across different subject orientations and robot positions, where the signal may be reflected or refracted before reaching the sensor, resulting in estimation errors. Figure 5 includes a grouped bar plot that shows the average error per position for each orientation, at one meter (green bars) and two meters (orange bars). The plot reveals that nearly all combinations exhibit deviations between the actual and estimated distances, with the largest discrepancies occurring on the negative side of the position, namely −5 to −1. This pattern is expected, as the robotic system approaches the subject from the negative positions (e.g., −5 to 0), where it first encounters the head. In this configuration, the radar has a limited surface area to detect, which reduces the strength of the reflected signal. In contrast, as the system moves beyond position 0, it gradually faces the torso and lower body, providing a larger and more reflective surface. Excluding Orientation 1, which consistently shows larger-than-expected distances across all positions, the other orientations tend to have average measured distances that are closer to or even smaller than the expected values. While this also constitutes an error, it results in the subject appearing closer to the radar, which may be due to the radar receiving stronger reflections from the torso or limbs in these orientations.

**Fig. 6:**
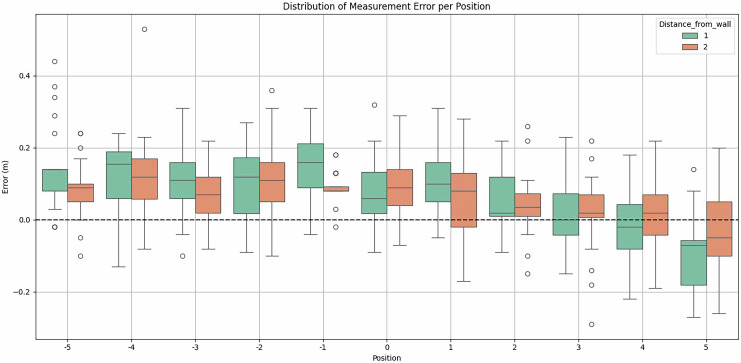
Distribution of measurement errors between annotated and Euclidean distances.

Figure 6 presents a boxplot illustrating the distribution of measurement errors of the annotated distances from the human annotator with the Euclidean distance that are shown on the Table 3, across different robot positions for the two measurement distances from the wall (1 m and 2 m). Each box represents the interquartile range (IQR), spanning from the 25th to the 75th percentile of the error values, capturing the middle 50% of the data. The horizontal line within each box marks the median while whiskers extend to the most extreme data points within 1.5 times the IQR. At the same time, values outside this range are shown as individual dots and considered outliers. A dashed horizontal line at 0 m denotes the ground truth. The plot of Fig. 6 reveals a clear trend: the median errors in many positions, especially those beyond position 0, tend to fall below the ground truth line, suggesting a consistent underestimation of distance. This behavior is expected given the setup: as the robotic system, moves from negative to positive positions, it transitions from facing the head to observing the torso of the subject. The torso, being a larger and more reflective surface, generates stronger returns that interpret the object as being closer than it actually is. Additionally, measurement at 1 m appears generally more stable (narrower boxes and fewer outliers) than at 2 m, highlighting that the radar performs more consistently at shorter distances. The wider IQRs and more numerous outliers for 2 m sessions suggest increased variability and possibly reduced accuracy in more distant measurements.

## Technical Validation

To validate the quality of the proposed dataset, we developed two distinct victim detection models: a Deep-Learning (DL) model, specifically a Convolutional Neural Network (CNN) and a Machine-Learning (ML) model, specifically a XGBoost model^18^. The results presented in this Section demonstrate the applicability of the dataset but also supports the motivation for its development. Indeed, our goal is to provide both a solid benchmark and a practical starting point for the research community, enabling others to further refine the dataset or extend it through novel data generation or model development strategies without the need for new data collection.

### Experimental setup

All experiments were conducted on a workstation running Ubuntu 24.04.3 LTS, equipped with a 12th Gen Intel® Core™ i7-12700F processor, 32 GB of RAM, and an NVIDIA GeForce RTX 4090 GPU with 24 GB of VRAM. Considering the dataset size and the additional memory overhead associated with preprocessing, data loading, and training, we recommend the use of a GPU with at least 24 GB of VRAM to ensure stable and efficient training. A minimum of 32 GB of system RAM is also recommended to support dataset handling and preprocessing operations.

Regarding storage requirements, a minimum of 50 GB of available disk space is recommended to accommodate dataset preprocessing, intermediate files, and potential data augmentation. In our experiments, which involved preprocessing without augmentation, total storage usage remained below this threshold.

### Data preprocessing

A pre-process step is necessary to enhance the quality of the data to be used as model input for training. As a starting note, only the magnitude of the complex envelope of the signal is used in this Section, as it sufficiently captures the relevant temporal and spatial variations of the signal for the needs of our analysis. Then, each radar data session is divided into overlapping samples, each comprising 470 frames (rows), corresponding to approximately 25 seconds of data (as in Fig. 2), with a 50% overlap between consecutive samples. To mitigate the influence of the direct path between the radar’s transmit and receive antennas, the first 1.44 meters of each sample are excluded from further post-process - with a range resolution of 5.12 cm (see Table I), this corresponds to the first 28 range indexes (i.e., columns, see Fig. 2). Consequently, each processed sample retains a dimensionality of 470 rows × 152 columns. Finally, DC removal is applied at each pre-processed frame to eliminate baseline drift.

### Model input data transformations

**Fig. 7:**
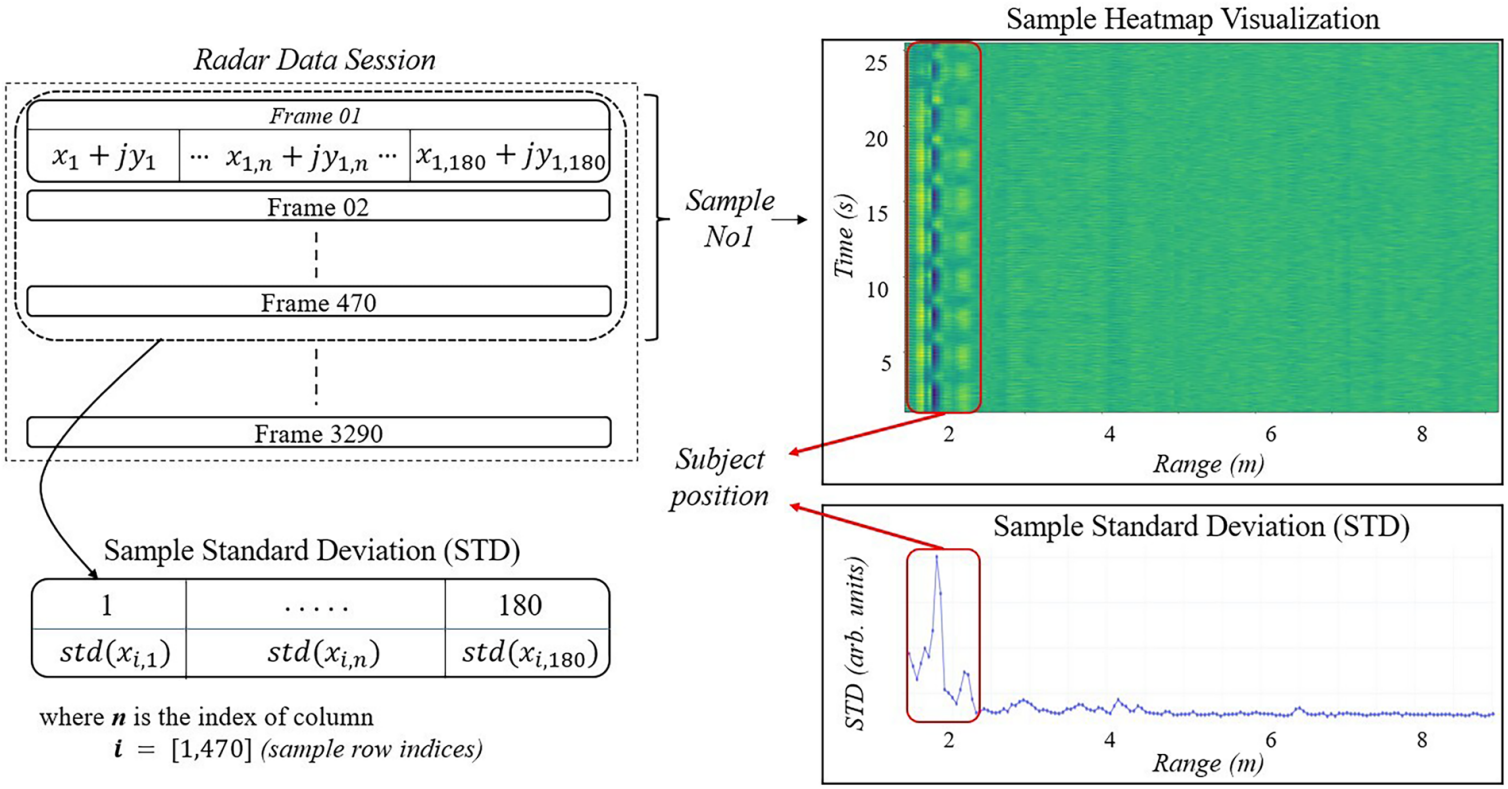
(**a**) Radar sample and column—wise standard deviation (STD) calculation (**b**) Visual representation of a radar sample signal amplitude heatmap (top) and a plot of the calculated STD vs. range (bottom).

In our current work, all data processing pipelines yield radar matrices with dimensions of 470 rows × 152 columns, corresponding to time steps and range bins respectively. This 2D format is ideal for convolutional neural networks (CNNs), which are designed to exploit both the temporal and spatial patterns present in data. However, this structure cannot be directly used in models that require one-dimensional input vectors, such as XGBoost^18^ or other traditional machine learning algorithms. To enable compatibility with these models, we compute the standard deviation (STD) across the 470 time-steps for each of the 152 range bins, effectively transforming the original 470 × 152 matrix into a **single 1 × 152 feature vector** per sample, as illustrated in Fig. 7. This transformation effectively reshapes the data into a 1 × 152 feature vector, capturing the variability of the signal in a compact form suitable for non-convolutional architectures. An example of that transformation is presented in Fig. 7(b): at the upper end there is a heatmap visualization of a random sample with an evident breathing pattern at ~1.62 meters. The corresponding STD across the 470 time steps of the sample is illustrated in Fig. 7(b)-bottom, with a distinct peak at the same distance of ~1.62 m.

It is important to note that this reduction alters the nature of the extracted information: while the original spatiotemporal structure can be used to detect periodic patterns like breathing, the STD transformation emphasizes signal fluctuations of any type, making it more suitable for capturing movement-based patterns. This trade-off reflects a shift in focus from detecting the respiratory analysis of the data to a broader analysis of the movement that happens on each sample.

### Victim detection – Classification of victim presence or absence

#### CNN architecture and parameters

**Fig. 8:**
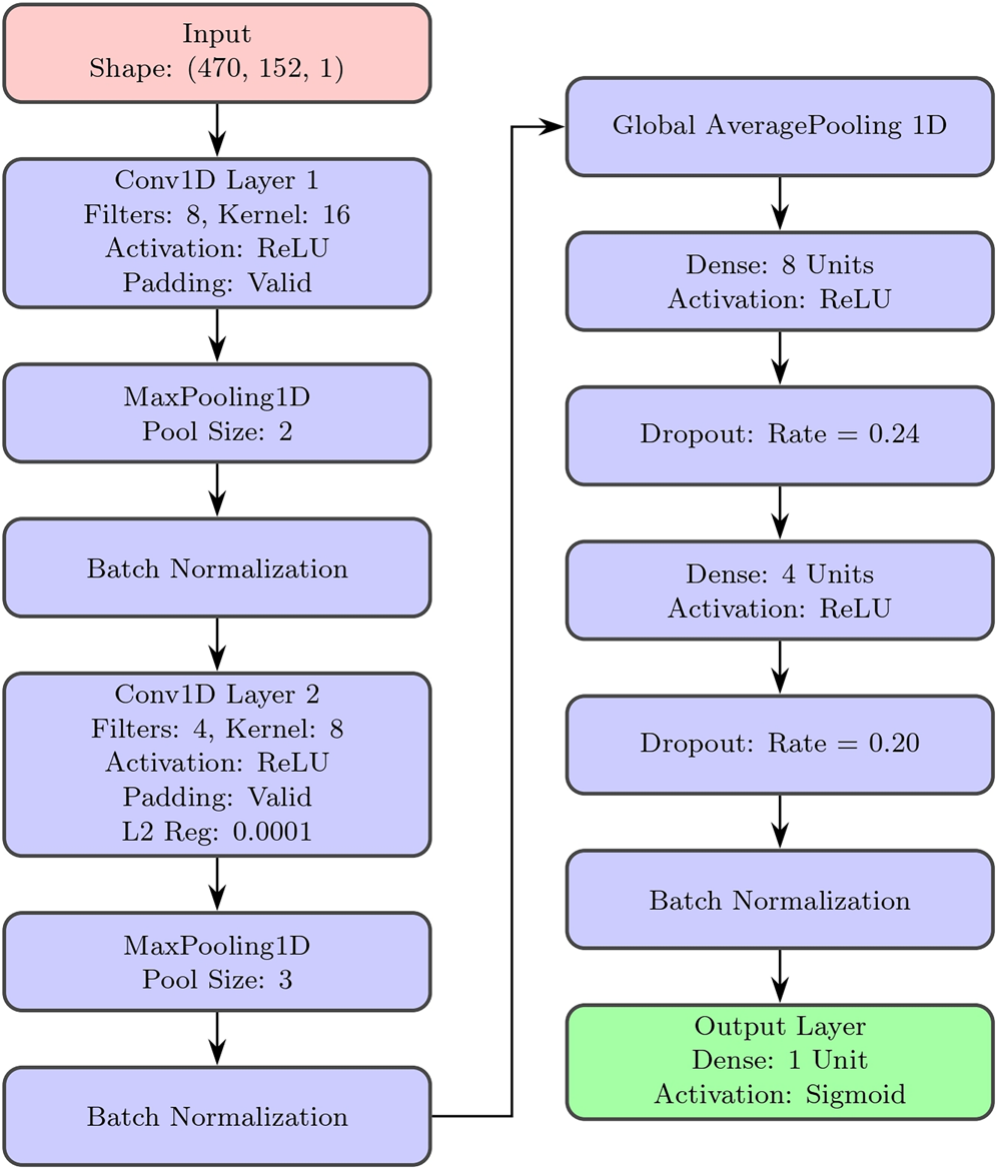
CNN architecture.

The primary architecture used for classifying human presence from the breathing pattern was a one-dimensional Convolutional Neural Network (1D-CNN), tailored to process radar signal matrices with dimensions of 470 rows and 152 columns. These dimensions represent the time evolution of radar returns across range bins, after excluding the first 28 columns to eliminate near-field reflections and direct path artifacts. Τhe CNN model began with a 1D convolutional layer utilizing 8 filters and a kernel size of 16. This was followed by a max pooling layer with pool size 2, batch normalization for stabilization, and a second convolutional layer using 4 filters with a smaller kernel size of 8. To prevent overfitting, L2 regularization was applied at this stage. The network further included another round of max pooling and batch normalization, then transitioned to a Global Average Pooling 1D layer, which was effective in minimizing distance-based bias. The resulting features passed through two fully connected layers with interleaved dropout operations before reaching a final dense layer with sigmoid activation for binary classification. A visual representation of the CNN that is constructed is illustrated in Fig. 8.

Training was conducted using the LOSO cross-validation methodology. In each fold, one participant was excluded from training and used exclusively for testing, thereby emulating real-world conditions where the system encounters an unseen individual. This strategy ensured robustness and generalizability of the proposed architecture. The remaining data were randomly shuffled to the training and validation sets with a ratio of 80-20% respectively.

### CNN results

The CNN model trained on the radar data demonstrated consistent and interpretable performance across all test subjects in the LOSO evaluation framework. The results after the LOSO cross-validation are presented in Table 4. Per-subject test results revealed high true positive rates and low false positive rates, particularly for cases where the subject was positioned centrally relative to the radar. False negatives were more frequent when the radar was offset from the subject’s torso, particularly at lateral robot positions (e.g., −5 to −3), which typically correspond to the subject’s head region with lower reflectivity. Additionally, the performance was more stable at 2 meters compared to 1 meter, a trend attributed to reduced near-field effects and better-defined reflection profiles. Notably, subjects N2 and N5 achieved the highest validation accuracies during training, while subject N1 showed the most favorable balance between training and validation metrics. The average macro precision achieved was 0.78, the macro recall was 0.80, and the macro F1-score was 0.77, as shown in Table 5. These results validate the effectiveness of the CNN architecture in detecting presence through breathing-induced radar fluctuations using unfiltered radar signals.

**Table 4:** Training LOSO Results for CNN.

Model	Training Accuracy	Validation Accuracy	Training Loss	Validation Loss
N1	0.889	0.739	0.316	0.523
N2	0.828	0.784	0.364	0.525
N3	0.858	0.719	0.304	0.637
N4	0.735	0.774	0.434	0.546
N5	0.751	0.807	0.444	0.560
N6	0.796	0.726	0.360	0.542

**Table 5:** Test LOSO Results for CNN.

	Precision	Recall	F1-Score
Macro Avg	0.78	0.80	0.77
Weighted Avg	0.82	0.78	0.78

### XGBoost architecture and parameters

Although not the primary focus of this study, a traditional machine learning model was implemented for comparison purposes. Using the same raw radar dataset as in the CNN pipeline, we extracted a one-dimensional feature vector by computing the standard deviation across time for each of the 152 range bins, resulting in a 1 × 152 input array per sample – as analytically discussed in subsection 4.2 above. This approach emphasizes amplitude variations over time, typically associated with subject movement or respiration.

The selected model was XGBoost, a gradient boosting algorithm known for its robustness and high performance in structured data classification tasks. The final configuration used a learning rate of 0.3 and a maximum tree depth of 6, all other hyperparameters where left on the default parameters from the creators of XGBoost^18^. The dataset was split using LOSO cross-validation strategy, in alignment with the CNN evaluation protocol, to ensure comparability.

### XGBoost results

The XGBoost classifier trained on raw data achieved results broadly consistent with the CNN model. Across the LOSO folds, the macro-averaged F1-score was 0.80 and the weighted F1-score was 0.83. Precision and recall metrics remained balanced, with slightly higher precision in detecting true presence cases and acceptable performance in identifying absence. The results can be found on Table 6.

**Table 6:** Test LOSO results for XGBoost.

Class	Precision	Recall	F1-Score
Macro Avg	0.86	0.78	0.80
Weighted Avg	0.86	0.84	0.83

Although this model does not leverage the spatiotemporal patterns captured by convolutional layers, its simplicity and explainability offer benefits in real-time or embedded applications. Nevertheless, the CNN architecture consistently outperformed XGBoost in scenarios with ambiguous or low-amplitude respiratory signals, highlighting the advantage of deep learning in extracting complex radar features.

### Distance estimation

Since the radar-to-target distance information is also critical for SAR operations, and to assess the quality of the dataset in this respect, we herein establish a baseline for distance estimation by employing a rule-based method using the STD across signal samples. Specifically, for each data sample, we identify the range bin with the highest STD as the bin that is most likely to correspond to the subject’s location. This rule is based on the consideration that increased signal variation due to victim movement (breathing or other micro-movements) will be captured by increased STD at the corresponding range gin. This approach efficiently serves as a form of movement detection, as it identifies the distance at which the signal exhibits the greatest fluctuation.

Consequently, the distance estimate for each sample was estimated using the following equation:

1 $$d=\left({i}_{max}\ast \varDelta r\right)+{d}_{{offset}},$$

where ${i}_{\max }$ is the index of the range bin with the maximum STD, $\varDelta r=0.0512\,{\rm{m}}$ is the range resolution according to Table 1, and ${d}_{{offset}}=1.44\,{\rm{m}}$ is a constant offset that accounts for the preprocessing steps of Section 4.1.

The accuracy of the distance estimates is evaluated via three performance metrics: Mean Squared Error (MSE), Mean Absolute Error (MAE), and Root Mean Squared Error (RMSE) as defined in Eqs. (2), (3), and (4), respectively.

2 $${MSE}=\frac{1}{N}\mathop{\sum }\limits_{i=1}^{N}{\left({j}_{i}-\hat{{i}_{i}}\right)}^{2},$$

3 $${MAE}=\frac{1}{N}\mathop{\sum }\limits_{i=1}^{N}|{j}_{i}-\hat{{i}_{i}}|,$$

4 $${RMSE}=\sqrt{{MSE}}.$$

The results shown in Table 7 demonstrate that the proposed method achieves reasonable accuracy, with a MAE of 0.49 m and an RMSE of 0.88 m, indicating that the system can estimate human distance with acceptable precision for coarse localization, particularly in the context of search and rescue (SAR) applications.

**Table 7:** Distance estimation performance results.

Performance Metric	Results
Mean Squared Error (MSE)	0.77 m^2^
Mean Absolute Error (MAE)	0.49 m
Root Mean Squared Error (RMSE)	0.88 m

## Usage Notes

To improve the usability and applicability of our dataset across varied research contexts, we herein propose a set of principled data augmentation techniques aimed at addressing class imbalance, as well as the development of a supporting software library. This library is designed to facilitate visualization and manipulation of the radar data, allowing researchers to efficiently apply filters, extract amplitude or phase information, and inspect signal characteristics in a reproducible and interactive manner. By providing these capabilities, the library enables faster experimentation and clearer interpretation of preprocessing steps, serving as a practical reference point for integrating our data into broader research workflows.

### Data augmentation and manipulation

The dataset exhibits a class imbalance, with samples indicating human presence outnumbering absence samples by a factor of one to five. To address this imbalance and improve the generalizability of trained models across diverse environments, one may apply a series of data augmentation techniques to the absence class as proposed herein. This augmentation is designed to synthetically increase the volume of absence data without introducing bias or distorting the intrinsic characteristics of the signals. This strategy not only improves the performance of the models but also encourages future work to leverage and extend the dataset through principled artificial data generation. This effect is reflected in the results obtained during the training of the models described in the present work.

The augmentation process includes a spatial shifting of the radar reflections, specifically by displacing the columns that represent object distances within a fixed window of ±20 columns. This adjustment ensures spatial variability while preserving the structure of the original signal. Following this, all signal values may be uniformly scaled by a factor of ±20% to introduce variation to the signal amplitude, simulating differences in sensor sensitivity, noise and recording conditions. To further augment the data, white noise may be added at a level corresponding to 20% of the mean signal value, reflecting sensor-induced fluctuations during acquisition. Finally, pink noise may be applied using a 1/f filtering approach to emulate natural environmental variations, as this noise exhibits more realistic spectral characteristics compared to white noise. This step is deliberately placed at the end of the augmentation pipeline to prevent it from being distorted by preceding modifications. Through this combination of techniques, an additional 5.5 hours of synthetic absence data were generated, effectively doubling the volume of available data for that class. These synthetic data are not included in the uploaded dataset^17^ but can be readily reproduced using the methods provided in the associated GitHub repository^15^.

The use of similar techniques for the presence class was tested, but did not ultimately implemented, with the goal of improving dataset generalization rather than direct data augmentation. Specifically, the column-shifting method described for the absence class was applied to the presence data to move the breathing subject within a random range. This approach effectively shifted the subject’s breathing from the original 1.5–3-meter range up to approximately 9 meters, near the radar’s range limit. This allowed the models to generalize better across positions, though it caused a small drop in performance at the original positions, which is expected due to the increased range of values and reduced sample density per location. This tactic can be more useful for conventional machine learning models compared to the CNN implemented in the current work, as the use of global pooling in CNNs reduces the need for coverage across all ranges.

### Radar data analysis toolset

Alongside the dataset, we also intended to create a toolset that allows users to easily interpret the collected data. To this end, we developed a dedicated library designed to assist users in extracting and processing the information contained in our dataset. This library is available under the GitHub page^15^.

This toolset/library enables prospective users to interpret the complex-valued radar data (amplitude and phase) by providing functions to extract the relevant components and perform basic preprocessing steps, such as data scaling or selecting only the desired time and distance ranges. Additionally, the toolset provides visualization capabilities, allowing users to generate single or multiple plots of the data, either as 2D heatmaps or as 1D plots of the standard deviation (STD) after applying the corresponding transformation functions.

Moreover, some of the most important features of the toolset include the implementation of multiple filtering methods, as well as support for advanced signal processing techniques such as Fast Fourier Transform (FFT), Wavelet analysis, and Doppler processing. These tools allow users to efficiently analyze, preprocess, and visualize the radar data, thereby facilitating both research and application development with the provided dataset.

## Data Availability

The dataset supporting this study, including all radar measurements, structured CSV files, folder organization, and metadata, is openly available on Zenodo under the title “A Comprehensive Dataset for Victim Detection in SAR Operations Using Robot-Mounted UWB-Radar Sensors”]^17^. It can be accessed at 10.5281/zenodo.16533267. The dataset includes both “Presence” and “Absence” classes, organized by subject ID, session, victim distance, victim orientation, and robot position, as detailed in the “Data Record” section of this paper. All files are distributed under the Creative Commons Attribution 4.0 International License (CC-BY 4.0).

## Code availability

All related code used in the present work is available in the respective GitHub page of our team^15^ and is offered as an open resource for use under the CC-BY-4.0 license.
